# When Intervention Meets Organisation, a Qualitative Study of Motivation and Barriers to Physical Exercise at the Workplace

**DOI:** 10.1155/2015/518561

**Published:** 2015-08-26

**Authors:** Thomas Viskum Gjelstrup Bredahl, Charlotte Ahlgren Særvoll, Lasse Kirkelund, Gisela Sjøgaard, Lars Louis Andersen

**Affiliations:** ^1^Institute of Sports Science and Clinical Biomechanics, University of Southern Denmark, Campusvej 55, 5230 Odense M, Denmark; ^2^National Research Centre for the Working Environment, Lersø Parkallé 105, 2100 København Ø, Denmark

## Abstract

*Objective.* To provide a comprehensive understanding of the motivational factors and barriers that are important for compliance with high-intensity workplace physical exercise that is aimed at reducing musculoskeletal disorders. *Method.* The present study, which used semideductive, thematic, and structured in-depth interviews, was nested in a 20-week cluster randomised controlled trial among office workers. Interviews were conducted with 18 informants with diverse fields of sedentary office work who participated in strength training at the workplace for 20 minutes, three times per week. Organisational, implementational, and individual motives and barriers were explored. *Results & Discussion.* The results show that attention should be given to the interaction between the management, the employees, and the intervention, as the main barrier to compliance was the internal working culture. The results emphasised the need for a clear connection between the management's implementational intentions and the actual implementation. The results emphasise the importance of ensuring the legitimacy of the intervention among managers, participants, and colleagues. Moreover, it is important to centrally organise, structure, and ensure flexibility in the working day to free time for participants to attend the intervention. Recommendations from this study suggest that a thorough intervention mapping process should be performed to analyse organisational and implementational factors before initiating workplace physical exercise training.

## 1. Introduction

Musculoskeletal disorders constitute a third or more of all registered occupational diseases [1, 2]. Back and neck pain are the most prevalent types of these musculoskeletal disorders and represent a major socioeconomic burden as a result of sickness absence compensation, disability pensions, and health services, among other factors [3]. Repetitive work, for example, computer use, is associated with such musculoskeletal disorders and pain [4, 5], and it can cause poor work ability [6] and sickness absences [3, 7–9].

Physical exercise is a cornerstone of health and wellbeing [10–12], and several studies provide solid evidence that targeted physical exercise is efficient in managing musculoskeletal pain that occurs in a work-related context [13–15]. Because a majority of adults spend many hours each week at work, the workplace, in contrast to population based physical activity interventions, offers a potential effective arena for targeting the adult population in general but also workers with musculoskeletal disorders, or other life style diseases. However, even if interventions are offered as a part of work, compliance with physical exercise can be challenging for employees. Studies show that, at best, there is moderate compliance with workplace physical exercise [15–17]. To improve workplace health promotion strategies, it is important to consider and act upon the factors that are associated with low compliance. A study of compliance to a workplace intervention showed that individual factors, such as self-efficacy [18], may not fully explain the low to moderate compliance with workplace interventions [19]. This is supported by research showing that individual psychological factors only partially explain adherence to physical activity [20, 21]. These and other studies emphasise and support social, cultural, and environmental factors as important for adherence to health behaviour [12, 20–22]. Furthermore, studies emphasise the psychosocial work environment, social relations, the workplace organisational structure, and the design of the intervention as important for the initiation of and compliance with interventions [19, 20, 22–25].

A cluster randomised study in Denmark (VIMS) [17, 26], in which this qualitative study was nested, showed the positive effect of high-intensity strength training in reducing musculoskeletal pain in the shoulder and neck region in participants who were regularly compliant. To increase compliance, the researchers in this study attempted to motivate participants as a part of the intervention. The motivational focus of this study was the participants' work environment (see Section 3 for further description of the VIMS intervention) [26].

The study showed that strength training had a clinically significant positive effect on pain reduction in the neck, shoulder, and lower back regions for those who were compliant to the protocol. However, the study also experienced a large dropout rate and many participants had low levels of compliance [17]. Regular compliance, which was defined as at least 20 minutes a week during the 20-week intervention, was only achieved by 56% of the participants [17].

Although the abovementioned study particularly focused on motivation and compliance in the intervention process, the level of compliance revealed that focusing upon these issues was not sufficient. The VIMS study's motivational efforts focused in general upon creating user-friendly training environments. The moderate effect on compliance despite an enhanced focus on these motivational factors in the VIMS study provided the necessity and basis for exploring if other motivational factors for exercise at the workplace were of greater influence than training environments for compliance. These motivational factors from VIMS are the basis for analysis in this study. In future interventions that involve high-intensity physical exercise in the workplace, to ensure compliance and to conduct more impactful physical exercise interventions, and thereby, to obtain the full effects, it is important to analyse the fundamental factors that increase compliance and the reasons for noncompliance and dropping out.

## 2. Aim

The aim of this study was to provide a comprehensive understanding of the factors that were important for compliance with physical exercise training in the workplace in the VIMS study. The present study, which used qualitative interviews, was nested in the VIMS study [17, 26] for the post hoc exploration of previous reports factors related to motivation and barriers by enrolling participants who had been randomised to an exercise intervention group. The object was to investigate the importance of (1) organisational factors (e.g., workplace culture, managers, and colleagues), (2) the implementational factors of the intervention (e.g., type of exercise and role of the exercise instructors), and (3) individual factors (e.g., motivation) as the VIMS participants' motivation for and barriers to compliance.

## 3. Materials and Methods

In the VIMS study [17, 26], in a sample of 573 office-working participants, 476 participants were randomised to weekly physical activity in the workplace and 97 participants to a control group. All of the participants gave their written informed consent to participate in this study, which conformed to the Declaration of Helsinki and was approved by the local ethical committee (HC2008103). The 476 participants were cluster randomised into four training-groups that performed the same amount of exercise and repetitions per week, that is, an equal training volume, for a total of one hour per week for 20 weeks during working hours. The first group (1WS) trained for one hour, once a week, the second group (3WS) trained 20 minutes, three times a week, and the third group (9WS) trained seven minutes, nine times a week. Group 4 (3MS) followed the same program as the second group but received supervision only during the initial week. The intervention group performed specific strength training with five different dumbbell exercises for the neck, shoulder, and forearm muscles. The exercises were basic, simple, and designed to be possible to perform for both beginners and more routine exercisers. The exercises were front raise, lateral raise, reverse flies, shrugs, and wrist extension. For more information and illustrations see Andersen et al. [26]. To enhance motivation, the training facilities were placed close to offices to reduce transportation time. Efforts were made to make the facilities appealing through the use of, for example, bright colours, windows, fresh air, and instruction posters. The instructors of the three groups were present at least 50% of the training sessions and focused on positive and qualified training feedback to maintain and enhance motivation and to increase the effect of the intervention. The intervention group filled out a training diary for each session. Furthermore, they answered an e-mail-based questionnaire that included validated scales, for example, the International Physical Activity Questionnaire (IPAQ) [27, 28], Self-efficacy [29, 30], Stages of Change [30, 31], self-reported compliance, pain, and motivational issues. For example, pain was measured by a VAS scale that ranged from 0 to 10. Participants' physical capacity was tested at the baseline, after ten weeks and at a follow-up. The VIMS program was launched 12 months prior to this study. For more information, see [26].

### 3.1. Sampling

We conducted semideductive, thematic [32], and structured in-depth interviews with 18 informants selected from the study population of the original VIMS study [17, 26]. The informants came from six different workplaces that were situated in six different geographical areas in Denmark [33, 34]. Because former studies had used a 3WS group as the typical method to organise a workplace exercise intervention regarding its intensity, volume, and time schedule, a subsample of participants from this group was selected [17, 35, 36]. Analysis of sociodemographic factors, BMI, and pain measures showed no differences between the 3WS, 1WS, 9WS, 3MS, and reference groups [17, 37]. Within one week after the intervention was completed, the informants were contacted at work by telephone by one of three members of the VIMS research group and were invited to participate in an interview. Information-rich individuals, who were willing and able to express their emotions and attitudes regarding the intervention, were selected (purposeful sampling) [38]. In all, ten regularly compliant and ten noncompliant informants were chosen for the subsample. The number of informants was estimated to be sufficient to reach data saturation. We defined a regularly compliant participant as a participant who participated in both the baseline and the ten-week testing of physical capacity. A noncompliant participant either dropped out of the VIMS project altogether by e-mail or telephone call to the support hotline, or, according to their weekly training diaries, ceased training between weeks four and eight of the intervention. Two noncompliant informants dropped out before being interviewed, which resulted in 18 informants. It was estimated that interviewing the number of included informants could result in data saturation, so there was no further inclusion. The informants were informed about the overall aim, design, and voluntariness of the interview study and that it was possible to withdraw from the study at any time. All interviews took place in the informants' workplace during work hours, not later than three weeks after intervention.  Table 1 shows the basic demographics and musculoskeletal pain intensity of the study participants for the subsample, as well as for the whole study population. For further information about the study group (e.g., demographics and compliance to intervention), see [17, 37].

### 3.2. Interview Guide

Questioning was purposefully and thematically designed [32] to elicit responses in organisational, implementational, and individual categories. These main thematic categories were chosen in advance on the basis of former studies that had elucidated these areas as relevant for further exploration [19, 20, 22–25, 39]. On the basis of the main categories, the interview guide was divided into specific themes concerning organisational factors (e.g., workplace culture, managers, and colleagues), implementational factors (e.g., type of exercise and the instructor's role), and individual factors (e.g., motivation, barriers and expectations). To explore each theme in more depth, we phrased specific questions to work as a base for the thematic exploration (e.g., see Table 2) [34]. See Appendix 1 in Supplementary Material available online at http://dx.doi.org/10.1155/2015/518561 for the interview guide.

The interview guide was developed by the first, second, and third authors of this paper. Agreement on the main categories and working questions was reached by consulting the literature, discussing the specific organisational structure of the workplace, and the specific implemental elements of the intervention. Three of the authors (BTVG, SCA, and KL) conducted all of the interviews and none of the informants had any preknowledge of these interviewers. The interviews were digitally recorded and the average duration of the interviews was 30 to 45 minutes.

### 3.3. Transcribing, Condensation, and Coding

All of the interviews were transcribed ad verbatim using an adapted transcription guideline [34, 40] and QSR Nvivo version 8.0 was used for the analysis of the 18 transcriptions.

Coding upon transcription followed a node tree related to the three main categories (organisational factors, implementational factors, and individual factors) and explored both motivational factors and barriers (Table 3). The organisational factor category included colleagues, organisation of work, and priority of time. The implementational category included the specific training, exercise instructor, and inspiration for physical activity within and after working hours. The individual factor category included reasons to participate, expectations, reasons for compliance, commitment, and feelings of guilt (see node tree in Table 3 for examples).

Analyst triangulation was used to increase interrater reliability. Initially, the first and third authors attained consensus on the procedure for the coding of the data and the node tree to limit bias [33, 41, 42]. The first and third authors coded one interview jointly. During this process, agreement on the content of the node tree categories was attained. The coding was performed deductively as a purposeful thematic analysis following the three main categories [32]. The second author did all of the consecutive coding of the interviews with a subsequent validation by the first author. The validation consisted of the first author reading through interviews and the categorical coding. If the first author disagreed with the coding, the discrepancies were discussed with the third author until a consensus was reached. Finally, essential meaning, supported by anecdotal evidence, was extracted by meaning condensation [43].

## 4. Results

The results from the qualitative interviews are presented in thematic order in the following sections: (1) organisational perspectives, (2) implementational perspectives, and (3) individual perspectives. See Table 4 for a conceptual map of the main categories, subcategories, and themes.

### 4.1. Organisational Perspectives

The first object of this study was to analyse how the informants perceived the organisational factors and how this perception influenced their motivation and barriers to compliance.

#### 4.1.1. Motivation

*(1) Support from Leading Authorities*. Most informants reported that the organisation had a great influence upon their participation in the intervention and their compliance to accomplish the specific exercises. One of the most important motivating factors experienced by a majority of informants was the acceptance of participation from leading authorities in the company, which gave legitimacy to participation in the intervention. By the leading authorities showing interest in the results and process and thus legitimizing the intervention through meetings and information, these informants felt that their participation was accepted. This acceptance improved informants' motivation for participation in the intervention.

*Regarding the intervention, we received information from the managers by e-mail. It is good to be encouraged to participate. Then, you know that the managers are interested in the results as well* (female, 37 years, compliant).

*(2) Flexibility in the Job Planning*. In general the employees at the workplace have very different flexibility in job planning making it easy for some and difficult for others to participate in the intervention. Informants indicating more flexibility in job planning state that this flexibility keeps them motivated and makes it possible for them to exercise 20 minutes a day during working hours. Furthermore, the structure of the intervention with three times 20 minutes of exercise makes it possible for those informants to schedule the exercise within an ordinary working day.

*Well, it is because it is three times, 20 minutes a week. It is easy to find 20 minutes during a working day* (female, 52 years, noncompliant).

*(3) Colleagues*. In general, a large part of the informants experienced a social change in the workplace after exercising because they met colleagues who were not a part of their daily working routines. This common frame of reference gave them something other than work to talk about and increased the social interface, which enhanced daily communication within the exercise group. Some informants described exercising with colleagues as a very important factor for their compliance. They reminded each other of the exercise bouts and, for this group, the small talk and laughter during exercise were the most important reason for compliance. The results indicate that social interaction between colleagues is important for compliance to the intervention but also to the social environment at the workplace in general.

*Those times during exercise where others attended, I met people and got to know them in a different way. You say hello in a different way when you have been lifting weights together. The social aspect is also what I like about this. I find it great to go to the exercise room and meet people I know, but never have an opportunity to talk to during working hours. It is great to have the exercise in common, I think. Maybe this was one of the main reasons for my participation* (female, 47 years, compliant).

#### 4.1.2. Barriers

*(1) Flexibility in the Job Planning*. In the same way as structure and flexibility in the job planning could be a possibility and a motivating factor for some informants, other informants indicated that lack of flexibility in the structure of their work was a major barrier. Moreover, approximately half of the informants had work tasks (e.g., meetings and teaching) outside of the workplace, and this absence from the workplace reduced flexibility and possibility to exercise and thereby introduced barriers to exercising.

In addition, barriers such as busyness, urgent tasks, deadlines, and unpredictability in the job (tasks that must be solved immediately, from hour-to-hour or day to day) were by some informants mentioned as barriers. These informants had the feeling that they were expected to make up for lost time after exercising and mentioned feeling stressed while exercising because job tasks were accumulating. The informants emphasised that, in many cases, they prioritised work to avoid getting too far behind.

*It is a barrier because we always have tasks waiting. There are always people knocking on your door, calling or asking questions. It is very difficult to “get away” from the office to do other things, to say: “Now, it is time to exercise”. There should be some big bells that call out to everybody, saying, “Now, it is time to do your exercises”, and then, it maybe would be easier *(male, 53 years, noncompliant).

As a consequence, when they chose to exercise, they hurried through the program in order to return to work. This did, in some cases, result in inadequate warm-ups and the incorrect execution of exercises, which sometimes resulted in inappropriate soreness. This pattern of action led, in some cases, to low compliance or dropping out.

*Maybe this has been the largest barrier. I have been a part of the group exercising three times 20 minutes a week, and sometimes, we did not take time to warm up properly. Then, you can feel a bit sore afterwards if you did not warm up before or stretch afterwards *(female, 37 years, compliant).

*(2) Guilty Conscience*. In opposition to the majority of informants experiencing their management as supportive other informants experienced a guilty conscience towards their employer when they exercised. The feeling of a wrong prioritisation was a major barrier for them. This often resulted in choosing work over exercise. They had the perception, as an implicit part of their working culture, that they must choose work before everything else. When these informants did not exercise because they prioritised work, they described having a guilty conscience towards the intervention and feeling guilty for not exercising after they had volunteered.

*Well, there is a need for some kind of change, an attitude change for people within these rigid systems, that it is acceptable, completely acceptable, to spend 20 minutes, three times a week, to prevent injuries. Maybe this is the real barrier. Sometimes, I had a really guilty conscience because I really could not find the time* (female, 52 years, noncompliant).

*(3) Colleagues*. Even though a large part of the informants found their colleagues motivating for the participation in the intervention, some informants also mentioned their colleagues as barriers. This relates to colleagues within as well as outside of the intervention group. These informants felt that having colleagues who were not part of the intervention put pressure upon them to keep working instead of exercising during working hours. This also relates to existing work culture as described above.

A few informants also stated that performing exercises and being sweaty in a public place at work were a barrier. Exercise facilities situated near areas that were heavily populated by colleagues (e.g., entrance and lunch room) were also expressed as barriers because there were colleagues who were watching.

Some informants stated that when a regular training partner ended the intervention, for example, due to busyness, holidays, or new a job, this was a major barrier that, in some cases, resulted in the other training partner dropping out as well.

*You do not want to stand near the entrance performing exercises when everybody else leaves. You get a lot of attention and remarks from colleagues who were passing by and were not part of the intervention. Sometimes I think: “Now, it is the time when people leave work”, then I do not want to exercise in front of everybody* (male, 53 years, noncompliant).

### 4.2. Implementational Perspectives

The second object of this study was to analyse how the informants perceived the implementation of the intervention and how this perception influenced their motivation, barriers, and compliance.

#### 4.2.1. Motivation

*(1) Reducing Physical Deterioration and Being Part of a Research Project*. As the VIMS intervention was a high-intensity physical exercise intervention that focused on exercises for the neck and shoulders, the informants were fully informed about the specific aim of the exercises. The information from the research group concerning the effect of the exercises motivated some informants to be compliant to the intervention. Other informants felt motivated to perform the efficient exercises because they felt they were able to reduce the physical deterioration inflicted by their job. For other informants, it was motivating to be part of a research project. They felt favourably towards the possibility of helping researchers to prevent future work-related injuries, and due to this, they delivered continuous and stable engagement. The informants emphasised that the weekly e-mail from the research team that was aimed at the awareness of pain and participation in the intervention helped them to keep their focus upon the intervention.

*“I would not say that it was fun, because you were not able to do any important stuff meanwhile. But it helps to keep the body in shape. It is necessary and it is what I have been doing too little of for all the years I have had sedentary work”* (male, 53 years, noncompliant).

The flexibility of the intervention design was also described by some of the informants as motivating because they were allowed to do the exercises whenever they could find time during their working day. 

*(2) Using VIMS Exercises as Inspiration*. Informants, who enjoyed the strength training in the intervention, saw VIMS as a chance to become stronger, while obtaining a visible result at the same time. These informants stated that they were inspired to do additional and various kinds of physical activities as a result of being a part of VIMS. A few informants who already engaged in strength training before the VIMS intervention stated that they implemented the new VIMS exercises in their own strength training programs.

*“I like that, and I also like to see, at the end, some muscles that I did not have before. In that sense, it is a nice visible result. I can also feel in my daily life activities that I am stronger. I like that very much”* (female, 37 years, compliant).

Some of the informants became motivated to engage in more physical activity. An informant described how he and his colleague hoped to continue the VIMS program once a week in the gym at the workplace. Another informant began to exercise with her whole family in the local fitness centre.

*I feel more motivated to participate in activities such as training and running in the fitness centre; activities that I actually dislike* (female, 33 years, compliant).

*We all started in the local fitness centre. We do strength training and it has helped me a lot. I do not have back pain anymore* (female, 45 years, compliant).

In general, the informants who were compliant were optimistic about continuing VIMS inside or outside the workplace after the intervention period. They hoped to combine VIMS exercises with other exercises. These informants experienced an increased awareness concerning VIMS exercises when they performed activities such as, for example, weight lifting, body pump, or even gardening, because they used the same muscle groups as those used in the VIMS.

*It (the VIMS exercises) is included in other activities that you do, like gardening or something in which you use—or feel that you use—the same muscles* (male, 53 years, noncompliant).

*(3) Introduction of Correct Techniques of Exercises and Enthusiasm of the Instructor*. The role of the intervention instructors was very important for the informants in VIMS. The majority of the informants agreed upon the importance of the instructors' initial introduction to the different exercises. These informants especially mentioned the importance of the instructors' introduction and coaching of the correct techniques for the exercises for their motivation. Furthermore, they expressed that feeling confident about the instructors' competence level influenced their motivation positively. During the intervention, the majority of the informants also found that the instructors' behaviour was motivating. Moreover, the instructors made them feel more secure due to their energy and enthusiasm when the informants participated, their guidance in the correct lifting techniques, or their adjustments to enhancing training efficiency. These informants also found it helpful to be able to ask the instructors other health-related questions concerning, for example, general physical activity and problems related to tension in their neck muscles.

#### 4.2.2. Barriers

*(1) Misunderstood Exercise Schedule and Inflexible Intervention Content*. A misunderstood perception by some of the informants that they had to follow a specific exercise schedule at specific times during the week was stated to be a barrier to compliance, and as a consequence, their compliance with the intervention, test, and retest was low. Moreover, approximately a quarter of the informants did not experience the VIMS design as being flexible, and they said that they were not aware of being allowed to exercise if the instructor was not present, which effected their compliance negatively. 

*(2) No Inspiration, Monotony, and Attention*. In contrast to the issues discussed in the motivation section, other informants also argued that the intervention did not influence their efforts or motivation to become more physically active in general. Moreover, the majority of the informants who were already physically active did not feel inspired to become more active.

Those informants not motivated by doing strength training mentioned other several barriers that arose from the implementation and the type of exercises in the intervention. These informants found strength-training exercises to be boring and they felt that fun aspects were missing during the training. The simple program, which only included five different exercises, was experienced as a very significant barrier because of its monotony. The informants felt that variety and whole body training were missing. They felt that the VIMS exercises should be implemented as a part of a whole training session that should include other exercises as well.

*But I think that if it is going to be implemented permanently, it needs more variety. I cannot tell what type of training it should be, but I know it will be boring and too predictable with only these five different exercises, and also it will be more acceptable to come up with excuses to skip the training* (female, 47 years, compliant).

Some of the informants were dissatisfied with the progression of the training. They felt demotivated when they could not perceive continuous progression. Other informants also felt obliged to exercise three times a week and felt that if they were not able to do it, the intervention was meaningless, and therefore, they lost the motivation to exercise. A few of the informants also felt obliged to continue their progression in the program despite an injury, which, in some circumstances, led informants to drop out of the program.

Finally, primarily the noncompliant informants described the program as unprofessional and static. They argued that the intensity of the training was too low and that no attention was paid to the individual aspects. Other informants felt that they were left alone. In general, they missed the attention and appreciation for their participation, and, for instance, a reminder or other types of support if their level of participation was too low.

*The only thing I wondered was (*⋯*) where were we relative to the project? When do we know something? I have no clue about how the participation rate is here. How many informants and so on? It would have been nice on a regular basis to get a feeling of—does the project still exist or not? Or do we just do something here all by ourselves?* (male, 53 years, noncompliant).

*(3) Competence and Behaviour of the Instructor*. In contrast to the majority of informants, a small part of the informants perceived the instructor as a barrier to their exercise. Some instructors were perceived as being nonpedagogical, lazy, and uncaring in their approach.

These informants argued their frustration if the instructors did not agree upon the execution of the exercises; for example, one instructor corrected their lifting technique although another instructor had said it already was correct. In the end, pressure from an instructor led to the drop-out of one informant because the instructor did not take the informant's physical capacity into account when increasing load in the exercises.

*Different instructors were not necessarily a bad thing. The important thing was for them to say the same things, agree upon the effort level and how to do the exercises* (female, 42 years, compliant).

*I dropped out simply because I felt I was not able to fulfill the demands of the instructor. He made it clear that if I could not do the exercises, maybe I was not suitable for the intervention. Even though I think I was not really bad, and surely, could have made progress if the weight had been increased slowly. But if I risked meeting an instructor later in the intervention telling me it was all wrong—I gave up, I must admit. I was angry and sad, and then I chose to quit* (female, 52 years, noncompliant).

### 4.3. Individual Perspectives

In this section, which concerns individual factors, the focus is on describing the primary individual experiences with the intervention. 

*(1) Pain, Positive Changes, and Social Activities*. In general, the informants described muscle pain as a primary individual motivation. A majority of the informants mentioned having various pain issues or seeing colleagues with pain as a motivation for participation. Pain in the neck and shoulder region, wrist, lower back, and head was mentioned as important. These informants hoped that the intervention would provide a future strategy to reduce and eliminate their pain issues. The majority of the compliant informants experienced reduced pain in their necks and shoulders and fewer headaches. An informant reported a rehabilitated wrist as a benefit from the intervention, and another reported a reduction in lower back pain.

*It is because we are sitting so much, that we are experiencing back pain, all of us. I see a lot of colleagues having pain in their arms and shoulders, and sooner or later, it will be me* (female, 45 years, compliant).

For some of the informants, the motivation for participation was free training, the hope to reduce the number of sick days and a reduction in expenses for physiotherapy and chiropractic care. Other informants also found it motivating and inspiring to leave work and engage in social activities with colleagues.

A majority of the compliant informants reported that they had experienced physical, psychological, and social changes during VIMS. The motivating factors that were mentioned included wanting to gain strength, better body posture, curiosity, learning new exercises, doing something good for oneself, feeling a “good conscience,” and having a “lightness” to the body. Furthermore, these informants reported having new experiences as a result of, for example, exercising to fatigue.

*After the introduction to VIMS, I have this clear goal to experience measureable physical results with a small effort of time within the workday* (female, 45 years, compliant).

#### 4.3.1. Informants Recommendations for Future Workplace Physical Activity Interventions 

*(1) Structure, Management, and Colleagues*. In general, the informants recognized that physical activity at the workplace had positive potential and that it was a very good idea. They acknowledged that it is difficult to be physically active after working hours. In general, they recommended that the physical activity should be implemented as a structured part of the workplace culture. The majority of informants emphasised the social aspect of physical activity as an important factor, and they found it beneficial to exercise with colleagues. Furthermore, the majority of informants stressed the importance of the participation of managers and directors to create the necessary legitimacy.

*It would be great to have 30 or 45 minutes of exercise, including a shower. I think the time would be well spent. Especially if the directors and managers would be the first to leave their work to exercise and then announce—“Now it is time to exercise”—then, it surely would be possible to find the time to exercise* (female, 37 years, compliant).

Some of the informants found it difficult to notify the managers of very heavy workloads, and at the same time, spend time exercising. Moreover, other informants found it problematic to be interrupted by exercise during their work tasks. In relation to this, the informants in general recommended that exercise should be scheduled in the morning or before the working day ends. They also recommended that the physical activity should be an active break during the day.

*I think it is positive to be forced away from your tasks, to get a break and to do something physically active. Then, you get away from your screen and get some fresh air through the system. I really think it is positive* (female, 52 years, noncompliant).

*In relation to work, I think you are more relaxed and awake when you return to your seat, and possibly, also more effective* (female, 42 years, noncompliant).

## 5. Discussion

This study explored the organisational and implementational issues in the high-intensity physical exercise program VIMS, which is concerned with musculoskeletal disorders [17, 26], because previous research had emphasised that, alone, it is insufficient to address individual factors for employees to adhere to high-intensity physical exercise at the workplace [18–21].

### 5.1. Organisational Perspectives

The main insight to be gained from this semideductive and thematic qualitative study was that a focus upon organisational factors within the workplace was decisive to attain high compliance, and thereby, to achieve a more effective intervention. The results show that attention should especially be given to the interaction between the management of the workplace, the employees, and the intervention since the management is both seen as facilitating and a barrier. This is because a main barrier to compliance among some informants was the internal working culture in which managers and colleagues signaled low priority of physical exercise and the intervention, despite having initially approved of participation in the intervention. The results emphasised the need for a clear connection between the implementational intentions of the management and the actual implementation. To avoid noncompliance in relation to this, the results show that it is important to ensure the legitimacy of the intervention among the managers, participants, and colleagues. Moreover, there is a need to centrally organise, structure, and ensure flexibility for all employees during the working day to allow time for the participants to attend the intervention. These results are supported by studies showing that work pressure, task demands, and support are important prognostic factors for compliance with workplace physical exercise interventions [25, 39, 44]. One of these studies stresses the fact that failing to overcome these factors in order to exercise for 20 minutes, three times a week, may not be explained by work demands, but rather, as a negative reaction to perceived and signaled work demands by managers [25]. The same picture is seen in the present study, in which differences in perceptions of manager support and work load affected the informants' compliance.

These results are also supported by the results of another study that analysed a high-intensity physical exercise intervention among laboratory technicians [45]. The results from that study emphasise the type of work and routines in the laboratory as important factors for compliance. In that study, work tasks were scheduled ahead of time, which made it easier for the employees and their managers to plan their high-intensity physical exercise. Moreover, the laboratory technicians were a homogeneous group of workers with strong support from their managers in the organisation, which was shown to be an important factor for compliance [19, 45].

This was not the case for about half of the informants in the present study, who emphasised the following barriers: the lack of managerial support, an inability to plan work tasks, the acute accumulation of work, and a significantly varied work schedule outside of the workplace. The literature supports these findings by emphasising both the structural and functional aspects of social relations as important for behaviour [46]. Because the individual acts to gain acknowledgement and to attempt to comply with social norms and attitudes in order to be included within the social community, the norms and attitudes at the workplace are important factors for compliance [44, 47, 48].

Another main finding from the present study relates to colleagues in the workplace. The results emphasise that colleagues are motivating factors when they offer acceptance and a strong supportive network, but also, that they are barriers when they signal dissatisfaction with extra work pressure and discontentment from not participating in the intervention. Because a workplace intervention involves influence from colleagues, poor social relations among colleagues may affect compliance negatively, whereas colleagues exercising together could emphasise compliance positively [25, 44, 49].

This is supported by the literature, which indicates that social relations are of the outmost importance in influencing behaviour, and therefore, also compliance to high-intensity workplace physical exercise [50]. This also stresses the fact that, in designing and implementing high-intensity workplace physical exercise interventions, it is important to consider specific workplace circumstances related to work organisation and management support, and the attitudes, norms, and work rhythms of colleagues, to enhance compliance [49, 51]. This could possibly be accomplished through a thorough intervention mapping process [52].

### 5.2. Implementational Perspectives

The way the intervention was implemented also seemed to play an important role in compliance. The results indicate that emphasis upon effective exercises in a scientific intervention was not enough. Informants highlight in general that varying, motivating, and entertaining exercises were important for compliance, and furthermore, for adherence over the long term. Therefore, a better compliance in this study could possibly have been accomplished if the exercises had been varied, rather than using the same exercises throughout the study. This would also have offered a better opportunity for the participants to implement the VIMS exercises in their own exercise programs. Moreover, a majority of the informants emphasise the importance of flexibility in the program, which, in case of flexibility in their working schedule as well, could make it possible for them to be self-determinant with regard to time and planning; this has been shown in the literature to be influential for motivation [53, 54].

The results also showed the instructors as key elements in the intervention. This is supported by other studies [17, 35, 55, 56]. Although a substudy of the VIMS intervention [26] showed that instructor supervision had no additional effect on pain reduction [37], the results of this qualitative study point out that if instructors are used in interventions, it is important to ensure that they have the same level of competence, give the same instructions and corrections, are able to take individual differences into account, and signal their presence during exercise sessions. Otherwise, the instructors can be barriers as well. This is supported by other studies, which also show that the instructor is important for compliance [20, 57–59]. The results also showed that the instructor provided an impression of safety and security; however, they also had the potential to generate a feeling of dependence in the participants, which could limit long-term adherence after the instructors leave the workplace when the intervention is completed [20]. The gap between an intervention and self-initiated physical activity is a common barrier in health interventions, and it should be taken into account in the design of workplace interventions [20, 60].

### 5.3. Individual Perspectives

Concerning individual factors, the results emphasised that a lot of informants were motivated by the anticipation of pain reduction and by being a part of a professional research intervention. This is supported by, for example, the Health Belief Model, which emphasises risk reduction and outcome evaluation as facilitating the initiation of, interalia, physical activity behaviour [61]. Transparency, through the provision of regular information and feedback from managers in the company and the research team, was also important for motivation for the majority of informants. Ongoing information and feedback were emphasised as important factors for the informants, both as a motivation, but also as a signal of legitimacy for colleagues who chose not to be a part of the intervention.

### 5.4. Methodological Issues

The results of this study should be considered as providing in-depth knowledge on specific and important motivational factors and barriers for high-intensity workplace physical interventions that are concerned with musculoskeletal disorders and should be seen within the scope of qualitative research and as inspiration for future studies. Despite a small number of informants, who, in some ways, could introduce sample selection bias and contradictory statements, valuable knowledge about compliance and adherence has been gained from these individuals. We acknowledge that another group of informants (e.g., one of the other three training groups in VIMS or from another workplace) may have perceived the VIMS intervention differently and, thus, could add to the understanding of motivation and compliance in a high-intensity workplace physical exercise intervention. However, we believe that this study emphasises fundamental issues that are concerned with participants' compliance with workplace interventions, which makes the results of this study relevant for future studies.

## 6. Conclusion

Participation in physical exercise at the workplace is very sensitive to how the workplace meets the intervention. The organisation or workplace in which the intervention takes place can be crucial for compliance with and the effects of the intervention. This is not only because of the lack of support of managers or motivation in the employees, but also due to practical barriers that are related to the organisation of the work (e.g., work time, place of work) and the implementation of the intervention (e.g., exercises, instructor) that prevent the employees' compliance, and sustained and steady participation in the intervention. Rather than considering individual factors, such as, for example, personal experience with physical activity, the results of this study show the importance of considering “how the intervention meets the organisation,” because the interaction between the individual and the environment seems to be a stronger predictor of compliance than individual factors alone. Thus, several aspects of the psychosocial work environment should be considered when implementing exercise at the workplace. Because the organisational and implemental perspectives are modifiable through appropriate workplace intervention, this introduces the future potential to reduce the prevalence of musculoskeletal disorders through high-intensity physical exercise workplace interventions, using the combined knowledge of exercise physiology, and social and psychological factors.

As our study only included office workers with neck/shoulder pain, more research should be performed to determine the influence of organisational and implementational factors on the barriers and motivations for participation in exercise among different job groups.

## 7. Perspectives

Major differences exist between workplaces. The recommendations from this study suggest that there should be a thorough intervention mapping process that analyses the relevant organisational and implementational factors that are important for motivation and barriers before initiating any high-intensity workplace physical exercise interventions that are aimed at the prevention and rehabilitation of musculoskeletal disorders [52]. Thus, it is possible to influence and prepare essential partners (e.g., managers and employees) at the workplace and, thereby, to enhance the possibility of high compliance with and the optimal effect of the high-intensity workplace physical exercise intervention.

## Supplementary Material

Appendix 1: Interview guide illustrating general themes and questions of the interviews.

## Figures and Tables

**Table 1:** Demographics and musculoskeletal pain intensity of the informants in the qualitative study.

	3WS (subsample)		3WS		1WS		9WS		3MS		REF	

Females, %	71%		69%		62%		56%		58%		58%	

	Mean	SD	Mean	SD	Mean	SD	Mean	SD	Mean	SD	Mean	SD

Age	44.6	(9.2)	46	(10)	47	(10)	45	(10)	45	(11)	46	(10)
BMI	24.2	(4)	24.7	(4.3)	25.2	(4.0)	25.3	(3.7)	25.6	(3.8)	26.0	(4.5)
Neck pain last 3 months	2.5	(2.5)	3.1	(2.4)	3.3	(2.3)	3.1	(2.3)	3.2	(2.4)	3.2	(2.3)
Right shoulder pain last 3 months	2.2	(2.1)	2.3	(2.4)	2.2	(2.3)	1.9	(2.2)	2.0	(2.4)	2.0	(2.4)

**Table 2:** Examples of working questions that were a base for the thematic exploration.

Main categories	Examples of working questions

Organisational perspectives	How did your managers influence your participation? How did your working rhythm influence your participation? How did your colleagues influence your participation?

Implementational perspectives	What influence did the instructor have on your participation? Did you consciously choose to exercise with or without an instructor? How could the instructors improve their performance?

Individual perspectives	Why did you participate in the intervention? Why did you comply with the exercise? What barriers did you experience that affected your participation? Why did you feel like you did not have the time to exercise? How do you think the physical activity in the workplace could have been implemented?

**Table 3:** Node tree of the coding. Not all subcategories were used for this study.

Main categories	Subcategories
Organisational perspectives	Reasons for compliance
	Motivation from colleagues
	Colleagues as barriers
	Work facilitates participation
	Obligation, conscience
	Organisational barriers
	Time/priority

Implementational perspectives	Reasons for compliance
	Organisation of the exercise
	Organisation of the exercise rooms
	Intrinsic value of the exercise form
	The role of the instructor
	The instructor as a motivation
	The instructor as a barrier
	The VIMS intervention as inspiration for private exercise

Individual perspectives	Expectations for participation in VIMS
	Meet expectations
	Personal changes
	Reasons for compliance
	Obligation, conscience
	External motivation
	Time/priority
	The VIMS intervention as inspiration for private exercise
	Attitudes towards/recommendations for exercise at the work place

**Table 4:** Conceptual map of main categories, subcategories, and themes in the results section.

Main categories	Subcategories	Themes
Organisational perspectives	Motivation	(i) Support from leading authorities
		(ii) Flexibility in the job planning
		(iii) Colleagues
	Barriers	(i) Flexibility in the job planning
		(ii) Guilty conscience
		(iii) Colleagues

Implementational perspectives	Motivation	(i) Reducing physical deterioration and being part of a research project
		(ii) Using VIMS exercises as inspiration
		(iii) Introduction of correct techniques of exercises and enthusiasm of the instructor
	Barriers	(i) Misunderstood exercise schedule and inflexible intervention content
		(ii) No inspiration, monotony, and attention
		(iii) Competence and behaviour of the instructor

Individual perspectives		(i) Pain, positive changes, and social activities
	Informants recommendations for future workplace physical activity interventions	(i) Structure, management, and colleagues
