# Supplementary material for: When Intervention Meets Organisation, a Qualitative Study of Motivation and Barriers to Physical Exercise at the Workplace
Source: ScientificWorldJournal. 2015 Aug 26;2015:518561. doi: 10.1155/2015/518561 (PMC4563103; doi:10.1155/2015/518561)
Supplement: Supplementary file 1 — Appendix 1: Interview guide illustrating general themes and questions of the interviews. [file 518561.f1.pdf]

Appendix 1

Interview guide

Organisational perspectives

1) Why did you participate in the intervention?
2) Which factors made you compliant / non-compliant to the intervention?
3) Which barriers to compliance did you experience?
4) Should physical activity be implemented as a structured part of your working day?
5) How did your managers influence your participation?
6) How did your working rhythm influence your participation?
7) How did your colleagues influence your participation?

Implementational perspectives

1) What do you think of the exercises?
2) Did VIMS motivate you to continue with the same exercises after the intervention?
3) Did VIMS motivate you to do other kinds of physical activity after the intervention?
4) Could you imagine exercising in your own spare time?
5) What influence did the instructor have on you and your participation?
6) Did you consciously choose to exercise with or without an instructor? Why?
7) How could the instructor improve?

Individual perspectives

1) Why did you participate in the intervention?
2) Why did you comply with the exercise?
3) What barriers did you experience that affected your participation?
4) Why did you feel like you did not have the time to exercise?
5) What was your motivation to participate in VIMS?
6) What expectations did you have for VIMS and what goal did you want to achieve?
7) Has the intervention met your expectations? Why / Why not?
8) How do you think the workplace physical activity could have been implemented?
9) What personal changes did you experience during the intervention?

Appendix 1: Interview guide illustrating general themes and questions of the interviews
